# Ultrasonography of the Fasciae and Common Pathologies: The Game Changer

**DOI:** 10.3390/diagnostics15091180

**Published:** 2025-05-07

**Authors:** Carmelo Pirri, Nina Pirri, Veronica Macchi, Andrea Porzionato, Raffaele De Caro, Levent Özçakar, Carla Stecco

**Affiliations:** 1 Department of Neurosciences, Institute of Human Anatomy, University of Padova, 35121 Padova, Italy; veronica.macchi@unipd.it (V.M.); andrea.porzionato@unipd.it (A.P.); rdecaro@unipd.it (R.D.C.); carla.stecco@unipd.it (C.S.); 2 Department of Medicine—DIMED, School of Radiology, Radiology Institute, University of Padua, 35121 Padova, Italy; nina_92_@hotmail.it; 3 Department of Physical and Rehabilitation Medicine, Hacettepe University Medical School, 06100 Ankara, Turkey; lozcakar@yahoo.com

**Keywords:** deep fascia, superficial fascia, diagnosis, ultrasonography, examination

## Abstract

Ultrasound (US) is rapidly gaining attraction among physicians for the evaluation of fasciae. Unlike traditional imaging, which often lacks specificity of pain localization, US examination stands out as a versatile tool, capable of detecting both structural and functional information. This unique capability allows for a comprehensive assessment of fasciae—the intricate connective tissue essential for human biomechanics. US examination offers a multiparametric approach for the assessment of thickness, echogenicity, stiffness, deformation and shear strain. This comprehensive examination is invaluable for identifying fascial pathologies that may not be detected during physical examination. In this study, we render and discuss common/elementary lesions of the fascia.

## 1. Introduction

Using ultrasound (US) examination, provisional definitions have become a crucial part of outcome measurements in musculoskeletal medicine [1,2]. Owing to its several advantages, US allows for comprehensive/dynamic assessment in multiple planes, providing detailed visualization of soft tissues with remarkable anatomical accuracy [3]. Likewise, US has also become a preferred modality for evaluating fascial abnormalities. However, since US is operator-dependent, with the scarcity of robust data concerning its use in fasciae, the interpretation/comparison of relevant studies is quite difficult. Specifically, standardized scanning methods and universally accepted definitions for US-detected fascial pathologies are lacking. While musculoskeletal US is widely employed for the assessment of muscles and tendons, the role of fasciae has traditionally received less attention. This pictorial narrative review aims to address this gap and establish greater consistency and reliability, with refined definitions for fascial pathologies.

## 2. Materials and Methods

This study is a pictorial narrative review aimed at illustrating the ultrasonographic appearance of fascial involvement in common disorders. The methodological approach was designed to provide a comprehensive and educational overview of the most frequent fascial pathologies encountered in clinical practice. The primary objective was to synthesize relevant imaging findings, supported by a selection of representative, high-quality sonographic papers and images.

### 2.1. Literature Search Strategy

A targeted literature search was conducted to support the clinical relevance and accuracy of the pictorial content. The search was performed across five electronic databases: PubMed, Cochrane Library, Web of Science, Google Scholar and Scopus. A combination of Medical Subject Heading (MeSH) terms and free-text keywords was employed to identify studies related to ultrasonographic evaluation of fasciae in pathological conditions. The keywords and search terms included but were not limited to “Fasciae”, “fascial pathology”, “ultrasound”, “sonography”, “ultrasonography”, “ultrasound examination”, “musculoskeletal ultrasound”, “fascial involvement”, “soft tissue disorders”, “myofascial diseases”, “entrapment syndromes”, “inflammatory fascial disorders” and “fascial fibrosis”. Boolean operators (AND, OR) were used to refine the search.

### 2.2. Inclusion Criteria

The literature search was limited to peer-reviewed articles published in English from inception until February 2025. For the purpose of this pictorial and narrative review, specific inclusion and exclusion criteria were established to ensure that the selected materials were clinically relevant, representative and aligned with the educational objectives of the manuscript. Studies and clinical cases were considered eligible for inclusion if they described the ultrasonographic evaluation of fascial involvement in the context of common musculoskeletal disorders. In particular, the review included articles and reports focusing on sonographic patterns of fascial abnormalities, such as fascial thickening, fibrosis, edema fluid accumulation, entrapment phenomena or inflammatory changes. Eligible publications encompassed peer-reviewed reviews, observational studies, prospective or retrospective case series and pictorial essays, provided they contained original sonographic data.

### 2.3. Exclusion Criteria

Studies were excluded if they did not focus on the sonographic assessment of fasciae or if they lacked a clear description of fascial involvement. Abstracts, conference proceedings and non-peer-reviewed materials were excluded.

### 2.4. Study Selection

The selection of studies was conducted following a structured, multi-phase approach. After the initial literature search, all retrieved records were imported into Zotero reference management software, where duplicate papers were identified and removed. A preliminary screening of titles and abstracts was then performed independently by two reviewers (C.P. and N.P.) to assess their relevance to the scope of this review, specifically focusing on the ultrasonographic assessment of fascial involvement in common musculoskeletal disorders. Following the preliminary screening, the full texts of the selected studies were reviewed in detail to ensure compliance with the inclusion criteria and the objectives of this pictorial narrative review. Any discrepancies between reviewers were resolved by consensus, or by consulting a third (L. Ö.) and a fourth (C. S.) reviewer.

## 3. Results

### 3.1. Normal Ultrasonographic Appearance of Fasciae

**Figure 1:**
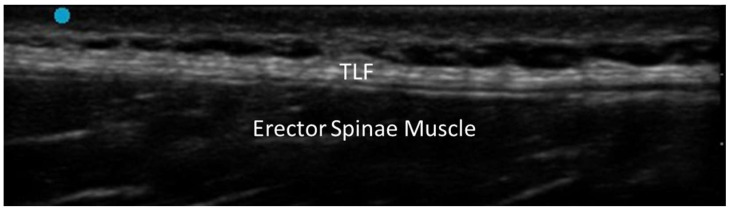
Normal US appearance of thoracolumbar fascia (TLF) of healthy subject.

Fasciae are continuous, fibrous connective tissue structures that envelop, support and separate structures (such as muscles, etc.) throughout the body. According to their anatomical location, fasciae are typically classified as superficial fasciae, which are located in the subcutaneous tissue, and deep fasciae, which surround muscles and neurovascular bundles. Functionally, fasciae play a critical role in force transmission, proprioception, compartmentalization and tissue sliding/movement. On conventional US examination, a normal fascia appears as a thin, regular, hyperechoic linear structure, located in the subcutaneous tissue (superficial fascia) and separating/enveloping muscles (deep fascia) (Figure 1).

Its appearance may vary slightly according to the anatomical region, depth and probe orientation. Dynamic US imaging of healthy fasciae reveals a smooth, gliding motion during active or passive movement of the underlying structures, without interruptions, adhesions or pathological thickening. There are accepted reference ranges of fascial thickness, reporting mean values ranging from 0.6 mm to 1 mm for superficial fascia and from 0.7 mm to 1.5 mm for deep fascia in the upper and lower limbs, depending on the anatomical site and patient-related variables such as age, sex and body mass index [4]. Color and Power Doppler signals may occasionally be detected in healthy fasciae when small perforating vessels cross fascial planes. The presence of persistent or diffuse Doppler signals within the fascial layers should be considered abnormal and suggestive of inflammatory involvement. Regarding sono-elastography, both strain and shear-wave have been explored as adjunctive tools to evaluate the mechanical properties of fasciae. In healthy conditions, fasciae exhibit high elasticity and low stiffness. However, current evidence is limited, and normative values for different anatomical regions have not yet been standardized.

### 3.2. General Pathologic Findings of Fasciae

#### 3.2.1. Fibrosis

Fascial fibrosis, characterized by the thickening and stiffening of fascial tissues, presents a significant challenge in clinical practice due to its complex pathology/etiology [5]. It is a pathological condition involving excessive deposition of collagen and other extracellular matrix components within the fasciae, leading to a loss of tissue elasticity and function [5]. It is associated with a variety of conditions, including chronic musculoskeletal pain syndromes, post-surgical adhesions and systemic diseases such as scleroderma [6]. Early and accurate diagnosis of fascial fibrosis is critical for effective management and improving patient outcomes.

**Figure 2:**
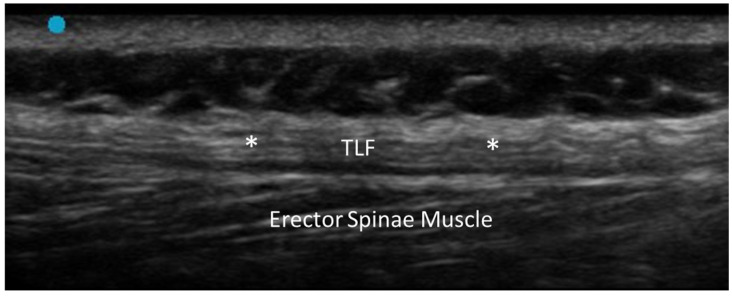
Fascial fibrosis of the thoracolumbar fascia. TLF: thoracolumbar fascia. *: areas of fibrosis.

On US, fascial fibrosis typically presents as a hyperechoic and thickened band of fascial tissue as opposed to normal fascia, which is usually characterized by the alternation of thin hypoechoic and hyperechoic lines. The increased echogenicity is due to the dense collagen fibers and reduced water content within the fibrotic tissue [5]. Dynamic US imaging can reveal restricted movement of the fascia relative to the underlying structures, a hallmark of fibrotic changes [6]. This restricted gliding is due to the stiffening/thickening of the fascial tissue, which can adhere to adjacent anatomical structures (e.g., muscle, tendon) as well. Doppler US can also be employed to assess the vascularity of fibrotic fascia. Typically, fibrotic tissue demonstrates reduced or absent vascularization due to the avascular nature of dense collagen deposits. However, in cases where fibrosis is accompanied by active inflammation, increased vascularity may be observed. In advanced cases, the fibrotic fascia my exhibit irregular or nodular contours, reflecting the heterogeneous nature of the fibrosis process [7,8] (Figure 2).

Shear-wave elastography (SWE) or strain elastography (SE) measures tissue stiffness and has been increasingly used in conjunction with conventional US to assess fibrosis. As confirmed by different studies, fibrotic fascia exhibits significantly higher stiffness values compared to normal tissue [9]. This additional information can aid in differentiating between various stages of fibrosis and in monitoring the disease progression or response to therapy. In fascial fibrosis, sono-palpation can help to reveal areas of abnormal stiffness and reduced elasticity, which may not be always apparent on static imaging alone [10,11].

#### 3.2.2. Densification

**Figure 3:**
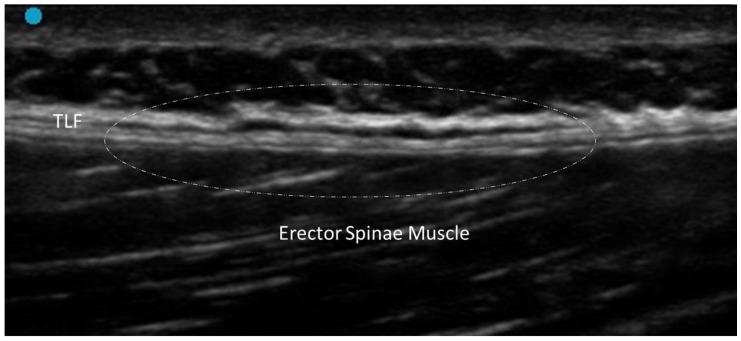
Fascial densification of the thoracolumbar fascia. Dashed-line circle: area of densification.

The term densification refers to an alteration in the sliding capacity of the fascial planes, while the general architecture of the fascia is preserved [5]. Fascial densification is a reversible condition—often linked to chronic overuse, trauma or local inflammation—for which accurate diagnosis is essential for effective treatment [12]. This alteration can only be assessed with dynamic examination [13]. Actually, the only finding on static US evaluation is thickening of the loose connective tissue around the fascia or between the fascial layers, suggesting an accumulation of fluid components [14]. It typically appears as area of decreased echogenicity, reflecting the accumulation of glycosaminoglycans (in particular hyaluronic acid) and an increase in water content [15,16,17]. US imaging also allows for a detailed evaluation of the fascial echotexture, offering insights into its density and elasticity (Figure 3).

Sono-palpation and dynamic assessment can detect subtle changes in tissue density and viscoelastic properties [16,17]. This is particularly important in cases where the densification does not yet present as a clear morphological change but has already affected tissue function. Dynamic assessment can highlight areas where the fascia demonstrates abnormal resistance or altered viscoelastic properties [12,13]. This can manifest as a reduction in the smooth sliding of fascial layers over one another, an essential function for normal biomechanical activity. Doppler US does not detect neovascularization but can help distinguish it from other inflammatory conditions.

#### 3.2.3. Scar

**Figure 4:**
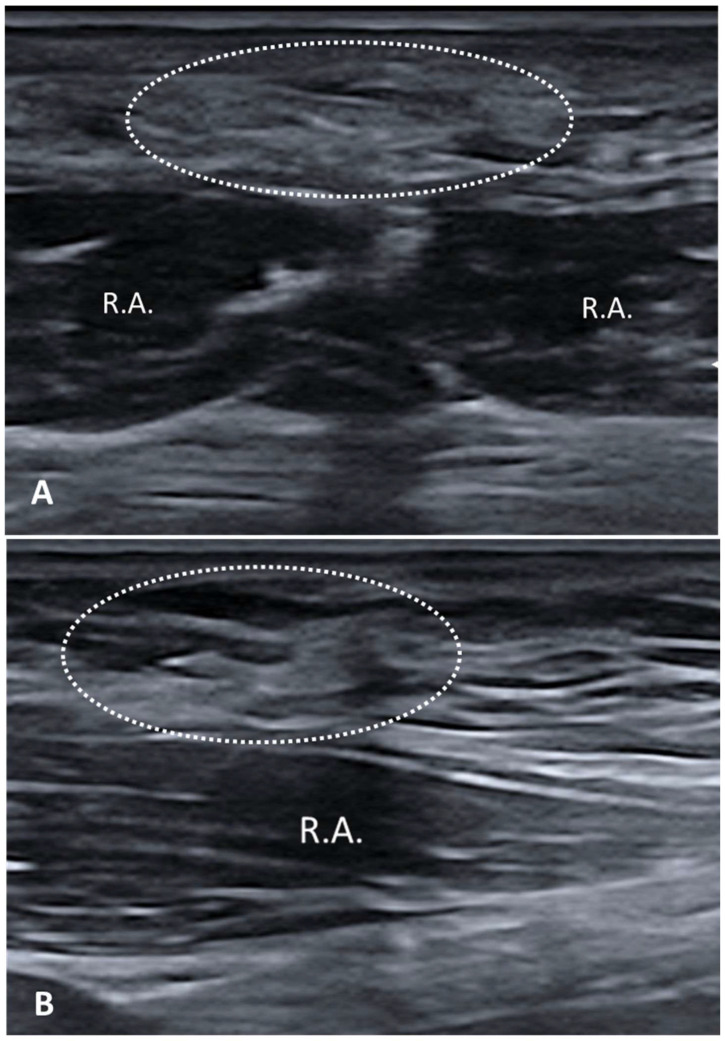
Scar tissue that envelopes the superficial/deep fasciae after cesarean section. (**A**) Scar tissue located along the midline (linea alba); (**B**) Scar tissue extending in the superficial and deep fascia over the rectus abdominis (R.A.) muscle. R.A. rectus abdominis muscle. Dashed-line circles: scar tissue.

Scars represent a dynamic biological process, reflecting the complex interplay of cellular mechanisms and extracellular matrix remodeling that are aimed at restoring tissue integrity yet often leave behind structural and functional compromises that can impact long-term outcomes. Assessment of the adhesions between fascial planes and the overlying skin is important for distinguishing different scar types [18,19]. US examination also clearly shows the different fascial layers, i.e., superficial fascia, deep fascia, superficial adipose tissue (SAT) and deep adipose tissue (DAT). This level of detailed insight helps to tailor interventions more effectively and to optimize the therapeutic outcome. For example, previous studies examining post-cesarean scarring demonstrated that while both the skin and subcutaneous layers remained unaffected, substantial thickening was observed in the deep fascia [18]. Such findings underscore the unique capability of US to provide a comprehensive evaluation of scarring, including the regeneration of fascial planes, appropriate layer thickness, proper fascial mobility and adequate vascularization [19]. Particularly hypertrophic and keloid scars, along with thickened or fibrotic fasciae, can significantly impact the quality of life by causing pain, stiffness and functional limitations. They appear as hyperechoic areas on US imaging, with varying degrees of thickness depending on the type and maturity of the scar [20] (Figure 4).

Hypertrophic scars and keloids usually present as thickened, hyperechoic bands with poorly defined margins, often accompanied by irregular internal echotexture, due to collagen deposition and fibrosis [21]. They often exhibit increased blood flow, which is a marker of active scar formation and can guide the treatment decision. The relationship between scarring and fasciae is complex, as scars can lead to fascial adhesions, fibrosis and thickening [18,19,20,21]. On US imaging, the fascial changes appear as hyperechoic bands within the fascial layers, often with a loss of the normal layered structure of the fascia. Fascial thickening and adhesions can restrict movement. Elastography can quantify the stiffness of the affected tissue, providing valuable information about the severity.

### 3.3. Inflammatory Conditions

#### Fasciitis

**Figure 5:**
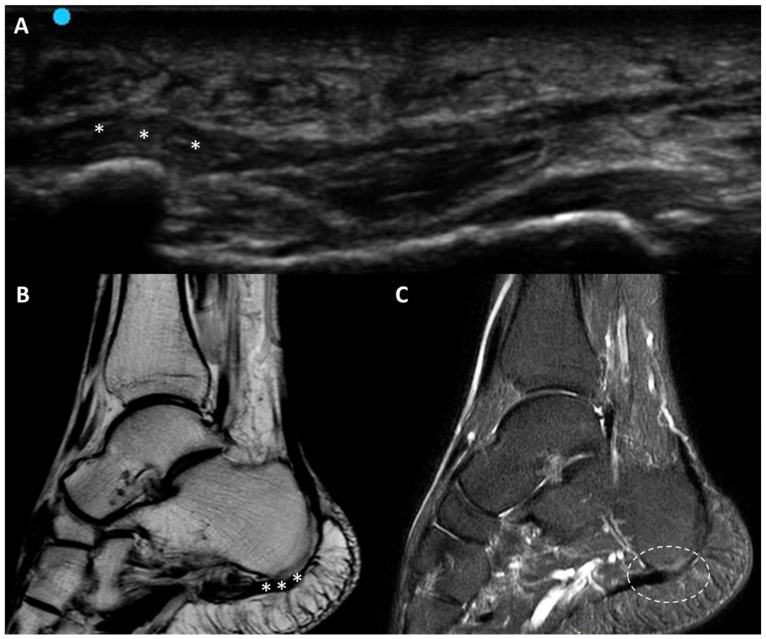
Plantar fasciitis. *: regions of increased plantar fascia thickness. (**A**) US examination demonstrating focal thickening and hypoechogenicity of the fascia. (**B**) Magnetic resonance imaging (MRI) using a dual proton–density turbo spin echo (DP_TSE) sequence, highlighting focal thickening. (**C**) MRI with a short tai inversion recovery (STIR) sequence, showing perilesional edema and signal intensity alterations consistent with inflammation.

Fasciitis encompasses a spectrum of inflammatory conditions, such as plantar fasciitis, necrotizing fasciitis and eosinophilic fasciitis. These conditions can arise from overuse, trauma, infection or systemic inflammatory diseases, leading to significant pain and functional impairment. On US, the affected fascia typically appears as thickened and hypoechoic compared to surrounding normal tissues [22,23,24]. For example, in plantar fasciitis, the plantar fascia often exceeds 4 mm in thickness, with associated hypoechogenicity reflecting edema/inflammation. Chronic cases may exhibit calcifications or bony spurs at the fascial attachments site, further highlighting the utility of US imaging in characterizing disease progression (Figure 5).

Dynamic US imaging can show the loss of normal fascial elasticity, with the affected tissue appearing stiff and less responsive to compression [25]. This type of examination is critical in distinguishing fasciitis from other sources of heel pain, such as fat pad atrophy or tendinopathy [26]. Doppler US may often reveal increased vascularity [27]. This feature helps differentiate fasciitis from other similar conditions like plantar fibromatosis or early fibrotic changes.

### 3.4. Tumours: Benign and Malignant

#### 3.4.1. Nodular Fasciitis

**Figure 6:**
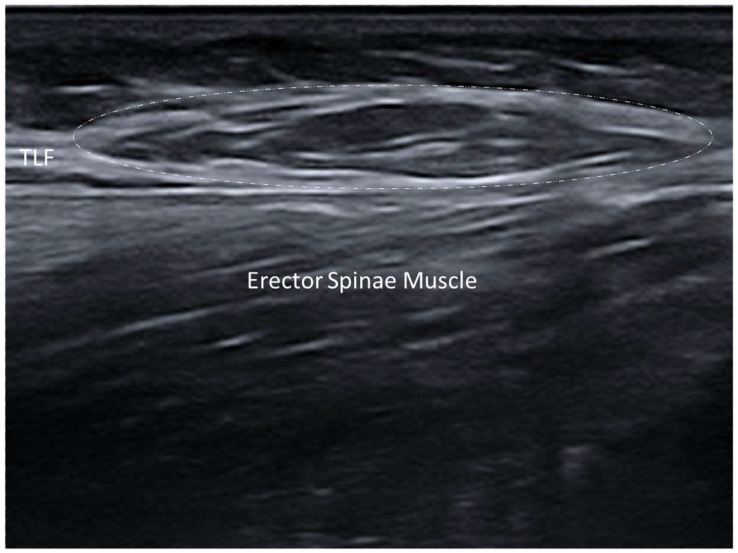
Nodular fasciitis of the thoracolumbar fascia. TLF: thoracolumbar fascia. Dashed-line circle: area of nodular fasciitis.

Nodular fasciitis is a reactive proliferative lesion of the fascia, presenting as a rapidly growing, painless mass [28]. Although benign, it may clinically/radiologically resemble malignant soft tissue tumors (e.g., sarcomas) and might therefore lead to diagnostic challenges. The etiology of nodular fasciitis is not fully understood, but it is often considered to be a response to minor trauma or inflammation. On US imaging, it appears as a well-defined, hypoechoic to isoechoic mass relative to the surrounding muscle tissue [28] (Figure 6).

The lesion is often superficial and may demonstrate a heterogenous echotexture with internal septations. In some cases, the mass may appear slightly lobulated, reflecting its benign and reactive nature. Unlike malignant tumors, nodular fasciitis usually lacks significant necrosis or hemorrhage, features that can be evaluated effectively with high-resolution ultrasound [29]. Doppler US might exhibit mild-to-moderate internal vascularity, which helps to differentiate it from highly vascular malignant tumors [29]. Overall, this vascular pattern, along with its well-circumscribed nature, would support a benign diagnosis and avoid unnecessary biopsy or surgical excision [30]. Similarly, elastography generally yields intermediate stiffness in contrast to firmer consistency of malignant soft tissue tumors [30].

#### 3.4.2. Elastofibroma Dorsi

**Figure 7:**
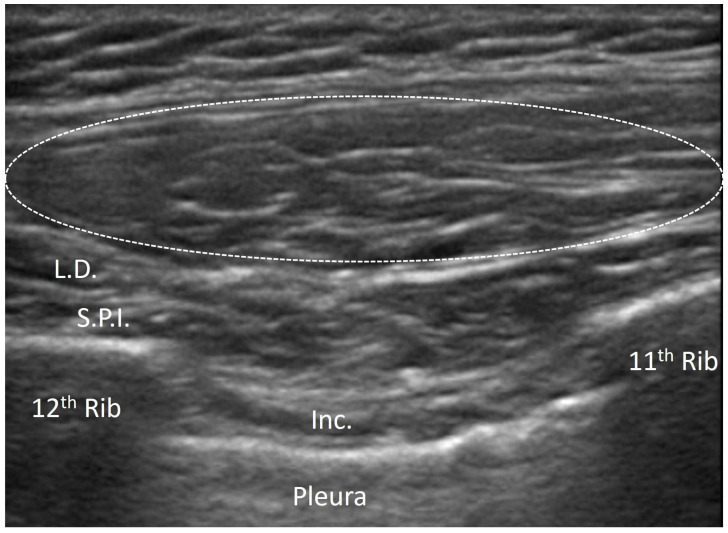
Elastofibroma dorsi of subscapular area. Dashed-line circle: the lesion appears as a well-defined, ovoid or lobulated intra-fascial mass with heterogenous echotexture. L.D.: latissimus dorsi muscle; S.P.I.: serratus posterior inferior muscle; Inc.: intercostal muscles.

Elastofibroma dorsi is a slow-growing benign soft tissue tumor, commonly located in the infrascapular region between the thoracic wall and the lower pole of the scapula [31,32]. Although it is typically asymptomatic, some patients may experience pain, discomfort or a snapping sensation with shoulder movements. On US imaging, elastofibroma dorsi appears as a well-defined, ovoid or lobulated intra-fascial mass with a heterogenous echotexture (Figure 5). The lesion often shows alternating linear hypoechoic strands, which correspond to the fibrous/fatty components of the tumor [31]. This “checkerboard” pattern is highly characteristic of elastofibroma dorsi and helps to differentiate it from other fascial and soft tissue tumors (e.g., lipoma or sarcoma) [32] (Figure 7).

One distinctive feature of elastofibroma dorsi is its tendency to occur bilaterally, and thus easy comparative imaging might again be the important advantage with US examination [32]. Doppler US might reveal minimal vascularity, reflecting its benign nature as well as differentiating it from more aggressive or malignant soft tissue tumors [32].

#### 3.4.3. Fibromatosis

Fibromatosis refers to a spectrum of fibroblastic proliferations that, while benign, tend to exhibit locally aggressive behavior with a propensity for recurrence after treatment [33]. These lesions can arise in various locations, including the abdominal wall, extremities and head/neck region, and can be associated with significant morbidity depending on their size and location [33,34]. Given the challenges in distinguishing fibromatosis from malignant soft tissue tumors, accurate imaging is critical for guiding management. On US imaging, fibromatosis appears as a hypoechoic mass with a homogenous echotexture. The lesion is usually well defined but can infiltrate the surrounding tissues, where it might have an irregular or poorly circumscribed appearance [33]. The homogeneity of the lesion, combined with its hypoechoic nature, helps to differentiate it from other soft tissue tumors, such as lipoma (which is usually more echogenic) or sarcoma (which may show a heterogenous echotexture due to necrosis or hemorrhage). On Doppler US, these lesions exhibit minimal-to-moderate internal vascularity, distinguishing them from more vascularized malignant tumors [35]. However, increased vascularity might be observed in larger or more aggressive fibromatoses (particularly those with rapid growth), reflecting active fibroblastic proliferation. One of the hallmark US features of fibromatosis is its tendency to infiltrate surrounding muscles, tendons, subcutaneous tissue, superficial and deep fasciae [35,36]. This characteristic finding is crucial for planning surgical interventions, as complete resection with negative margins is often necessary to reduce the risk of recurrence.

#### 3.4.4. Desmoid Tumor

Desmoid tumor refers to the fibroblastic proliferation within fasciae that, while histologically benign, can behave aggressively with a high risk of local invasion and recurrence [37]. It can arise in any anatomical location, but most commonly in the abdominal wall, extremities and head/neck. Given its potential to cause significant morbidity, especially when involving vital structures, accurate imaging is essential for prompt management [37]. On US imaging, it appears as a well-defined, hypoechoic mass with a homogenous or slightly heterogenous echotexture [38]. Its borders may appear well circumscribed or ill defined, depending on the degree of infiltration into the surrounding tissues. The internal structure of the tumor is usually homogenous, but in some cases, it may show areas of hyperechogenicity due to fibrosis or calcification [39]. On Doppler US, it often exhibits moderate-to-high internal vascularity, different from other benign soft tissue masses like lipomas. The vascular pattern can also aid in monitoring response to therapy, as reduced vascularity may correlate with a favorable treatment response [39].

#### 3.4.5. Lipoma

Lipomas are the most common type of benign soft tissue tumor, typically presenting as a slow-growing, soft and painless mass in the subcutaneous tissue. While most lipomas are superficial and encapsulated between the layers of superficial fascia, some may develop deeper in the deep fascia or between the fascial planes of muscles, interacting with or displacing the fasciae [40]. Understanding the relationship between lipomas and fasciae is critical for planning management strategies, especially in case where the lipoma causes pain or functional impairment due to the compression of fascial or neurovascular structures [17,40].

**Figure 8:**
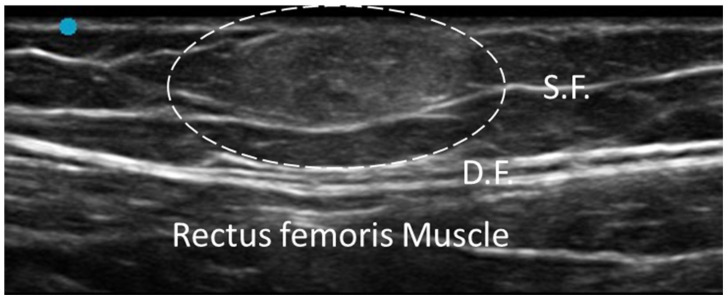
Lipoma enveloped by the superficial fascia at the middle third of the thigh (long axis). Dashed-line circle: lipoma. S.F.: superficial fascia. D.F.: deep fascia.

On US, lipomas appear as well-defined, homogeneously hyperechoic or isoechoic masses [41]. Their borders are usually smooth, and their shape is often oval or round. Because of their soft adipose tissue composition, they exhibit low internal resistance to sound waves, appearing as hypoechoic [41]. Superficial lipomas are most commonly located above the deep fascia, rarely infiltrating or involving its layers. In deeper lipomas, US examination can delineate their boundaries and assess any displacement of surrounding structures, including the superficial and deep fasciae [42] (Figure 8).

US is again contributory to the differential diagnosis. For instance, fibrolipomas may show internal septations whereas angiolipomas may display hypoechoic regions due to the presence of vascular channels. The absence of heterogeneous components, necrosis or infiltrative borders is key to confirming the benign nature of the lesion [43]. Dynamic US is not used in lipomas’ routine evaluation, as they are generally static and non-invasive. However, in visualizing their movement relative to the fasciae during muscle or joint movements, the absence of infiltration/compression onto deeper structures may be confirmed. Mapping the precise location would be paramount for surgical plans in case needed. Sono-palpation can be used to assess the consistency and compressibility of lipomas [15,17]. This approach often confirms the soft, compressible nature of the lipoma and the absence of resistance during manual compression, which is indicative of benignity.

Elastography is also a useful adjunct to B-mode imaging, whereby lipomas are generally soft and compressible, in contrast to firm masses like fibromatosis or malignant tumors [44]. On Doppler US, lipomas are typically avascular; however, larger ones may exhibit peripheral vascularity due to their size and proximity to blood vessels. The absence of substantial vascularity further supports their benign nature and differentiates them from more vascularized (malignant) tumors [40,41,42,43,44].

### 3.5. Musculoskeletal Disorders

#### 3.5.1. Myofascial Muscle Injury

**Figure 9:**
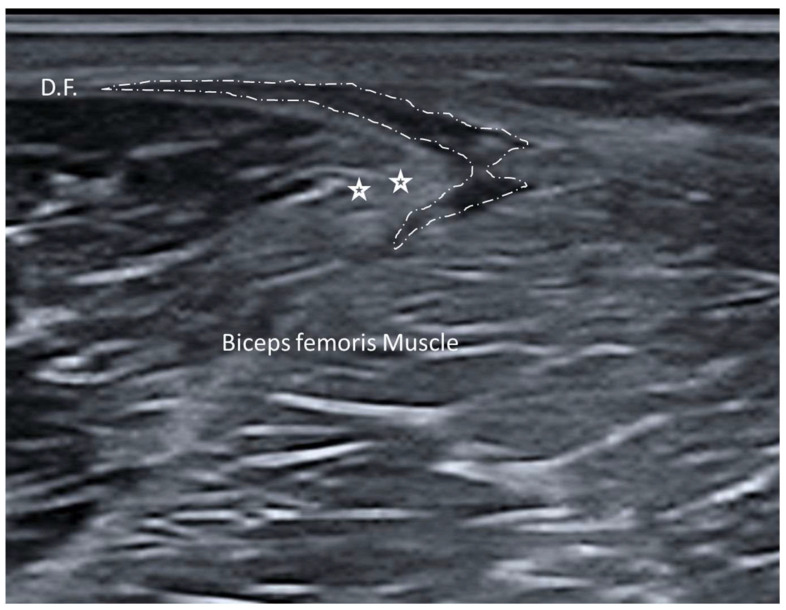
Myofascial muscle injury of biceps femoris and fascia lata. Dashed line: fascia lata injury with its delamination. D.F.: deep fascia, here fascia lata. Star symbols: regions of injury of biceps femoris muscle.

The myofascial unit, consisting of skeletal muscle and its surrounding deep fascia, is essential for efficient movement and optimal biomechanical function. Injuries to this unit, often termed as myofascial injuries, can significantly disrupt normal function [45,46]. The involvement of fasciae plays a critical role in both the onset and persistence of relevant symptoms. Yet, far from being a passive structure, fasciae actively contribute to muscle coordination, force transmission and proprioception [45,46]. US is often the first line of investigation in myofascial lesions, whereby tears are visualized as discontinuities within the fascia and muscle fibers, and depending on the severity, there may be fluid collection or hematoma surrounding the affected area [47,48] (Figure 9).

In cases of severe injury, the fascia may also be disrupted/torn, leading to thickening, irregularity or detachment from the underlying muscle. Dynamic US and sono-palpation can help pinpoint areas of injury and also mobilize fluid collection, if present. Elastography can differentiate between areas of normal muscle (less stiff) and fibrosis/scar tissue (more stiff and less elastic) [49]. On Doppler US, acute (not chronic) muscle injuries may reveal increased vascularity, i.e., inflammation [49].

#### 3.5.2. Morel–Lavallée Lesion (MLL)

This is a rare, post-traumatic, closed soft tissue injury caused by shearing forces that separate skin and subcutaneous tissue from the underlying deep fascia—creating a potential space filled with a mixture of blood, lymph, fat and necrotic debris [50]. MLL is most commonly found over the bony prominences (e.g., greater trochanter) due to shearing injury that disrupts the relationship between the subcutaneous tissue and the superficial/deep fascia and retinacula cutis. Clinically, these lesions may present as a fluctuant or firm mass, with skin discoloration, swelling and variable tenderness, often emerging days to weeks after the trauma [51]. If not diagnosed and treated in a timely manner, MLL can progress to chronic encapsulated collections, leading to fibrosis, secondary infections or persistent inflammation, complicating the treatment and recovery alike.

On US examination, fascial detachment and hypoechoic or anechoic fluid collection are often present, and the lesion contains internal debris such as blood or necrotic fat [52]. Chronic MLL may exhibit hyperechoic fibrous encapsulation within the space between the superficial and deep fasciae. Dynamic US examination further enhances diagnostic accuracy by evaluating fascial mobility and fluid shifting within the lesion [52,53]. Restricted movement in the fascial plane or the identification of adhesions can indicate a chronic/complicated lesion. Doppler US is utilized to detect peripheral hyperemia, which is indicative of inflammation or potential infection. It also helps to confirm the absence of vascularity within the lesion itself, differentiating MLL mainly from vascular malformations [52]. In acute lesions, elastography typically reveals lower stiffness due to the liquid nature of the collection, whereas in chronic lesions, increased stiffness is often present due to fibrotic changes and encapsulation [52]. Sono-palpation is an important dynamic modality used to assess compressibility and displacement of the lesion which provides valuable diagnostic clues. Of note, assessing the pain response during this maneuver can be helpful in evaluating the inflammatory state [16].

#### 3.5.3. Myofascial Trigger Points

A myofascial trigger point (MTrP) is a hyperirritable nodule located within a taut band of skeletal muscle. It is often referred to as a “muscle knot” and can cause localized and referred pain [53]. An MTrP may be active, causing spontaneous pain, or latent, becoming painful only when palpated. They are associated with muscle dysfunction, a reduced range of motion and underlying fascial abnormalities [53]. US examination has emerged as a valuable tool for the assessment of MTrPs, providing objective and reproducible insights into their structure and interaction with surrounding fasciae [53]. MTrPs, which are commonly associated with myofascial pain syndrome, appear as hypoechoic or hyperechoic regions of gray-scale US, often characterized by disrupted fascial continuity and altered muscle texture [53,54]. Fascial abnormalities, such as thickening, fibrosis and restricted gliding between fascial layers, are critical contributors to the persistence of MtrPs and can also be effectively visualized through US examination [52]. Doppler imaging further highlights vascular changes in the vicinity of active MTrPs, revealing increased vascular resistance and distinct blood flow patterns compared to normal muscle [55]. During elastography, MTrPs are stiffer than the surrounding tissue, with reduced vibration amplitudes reflecting heightened tension and decreased elasticity [53].

#### 3.5.4. Fascial Nerve Entrapments

**Figure 10:**
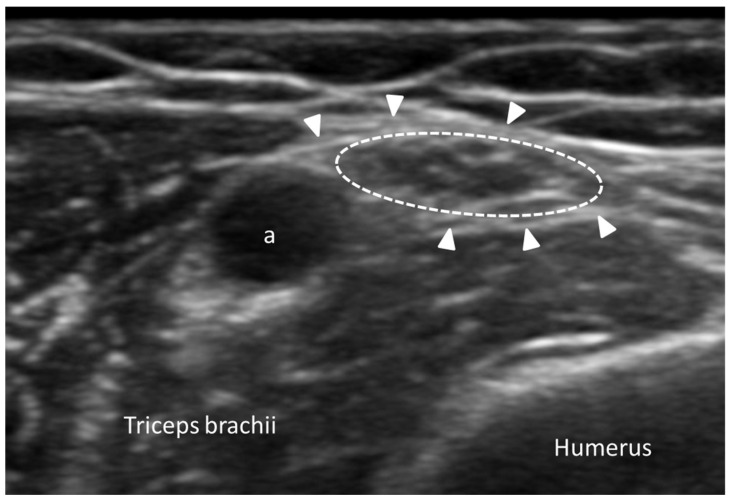
High-resolution ultrasound image illustrating the radial nerve (dotted circle) accompanied by deep brachial artery (a) immediately before entering the spiral groove. Arrowheads delineate the paranueral sheath enveloping the radial nerve.

Fascial nerve entrapments are neuropathic conditions in which peripheral nerves are functionally impaired due to altered mechanical relationships with surrounding fasciae, rather than classical compressive lesions [56]. The paraneural sheath, a specialized extension of the deep fascia, envelops the nerve and plays a key role in neurovascular protection, proprioception and the transmission of myofascial forces [57]. US examination, in particular in its high-resolution static and dynamic applications, has emerged as a powerful tool for evaluating these disorders. US enables (1) the precise visualization of the nerve’s morphology, echogenicity and cross-sectional area; (2) the identification of fascial thickening, loss of normal fascial planes or altered fascial tissue echotexture; and (3) the detection of perineural fluid, fibrosis or structural changes suggesting chronic entrapment. However, dynamic US adds crucial diagnostic value by assessing (1) the mobility of the nerve within and against fascial structure during passive and active movements; (2) the presence of abnormal gliding patterns, fascial adhesions or delayed nerve recoil; and (3) comparative analysis with the contralateral side to detect subtle asymmetries in movement and positioning. Dynamic scanning is particularly useful in cases with non-specific symptoms or negative electrophysiological tests, where fascial restrictions alter nerve function without clear structural compression [58]. Moreover, US examination allows real-time sono-palpation and sono-Tinel to reproduce symptoms and guide targeted interventions [16] (Figure 10).

#### 3.5.5. Compartment Syndrome

**Figure 11:**
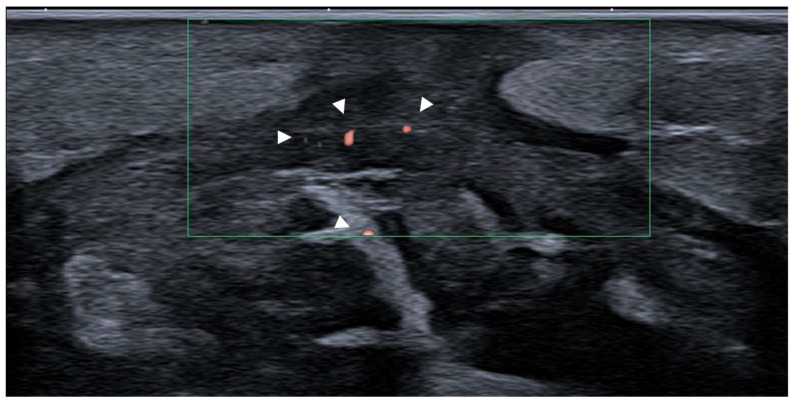
Post-fasciotomy US examination of fascia lata using superb microvascular imaging (SMI). Short/transversal axis scan of medial thigh demonstrating the fascia lata following surgical decompression. The fascia appears thickened, with a hypoechoic appearance suggestive of postoperative remodeling. SMI highlights low-flow microvascular signals within and adjacent to the fascial layers (arrowheads), consistent with ongoing neovascularization and tissue repair.

Compartment syndrome represents a pathophysiological cascade in which the fascial envelope plays a pivotal role [59,60,61]. The deep fascia defines the anatomical boundaries of each compartment and determines the capacity to accommodate volume changes. When intracompartimental pressure rises—whether due to trauma, hemorrhage or ischemia–reperfusion injury—the rigid fascial constraints impede venous outflow and capillary reperfusion, precipitating cellular hypoxia and tissue necrosis. In this context, the deep fascia is not merely a passive boundary but an active player in both its onset and progression. US examination, in particular with high-frequency linear probes, offers an unparalleled tool for the direct visualization of fasciae in real time. The deep fascia can be dynamically assessed for thickening, irregularity, loss of continuity or abnormal hypoechogenicity, which may reflect inflammatory changes or structural compromise. Moreover, interfascial fluid collections, hematomas or subfascial edema can be detected early, enhancing diagnostic accuracy [61]. In the postoperative phase, the fascial status remains clinically relevant. US examination enables the monitoring of fascial healing, detection of post-fasciotomy fibrosis or adhesions and assessment of persistent fascial constriction in cases of incomplete decompression [62]. In addition, Doppler US, in particular superb microvascular imaging (SMI), allows for the evaluation of vascular dynamics across fascial planes, providing insights into reperfusion and potential ischemic sequelae (Figure 11).

#### 3.5.6. Plantar Fascial Rupture

**Figure 12:**
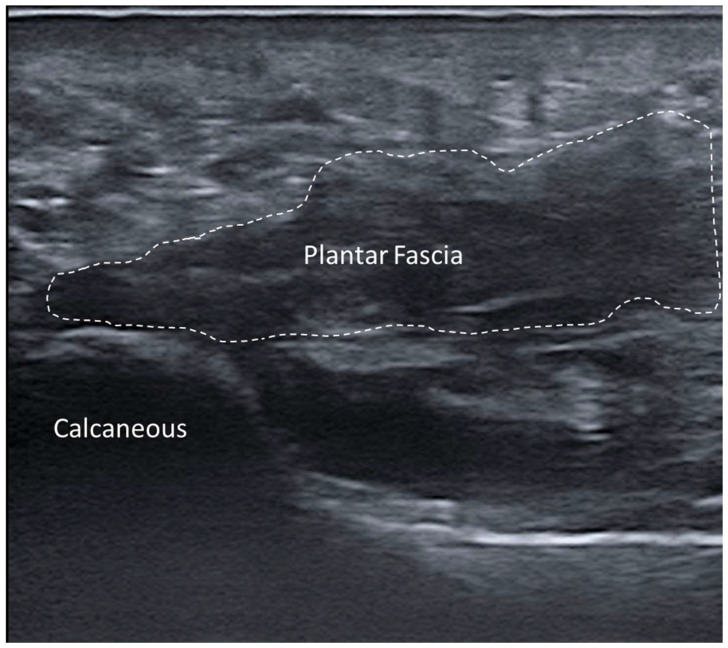
Plantar fascia rupture is seen as focal discontinuity in the hyperechoic, fibrillar architecture of the fascia, accompanied by hypoechoic areas indicative of edema or hematoma. Dashed-line: plantar fascia rupture.

Plantar fascia rupture presents distinct ultrasonographic features that are invaluable for accurate diagnosis and management. The hallmark finding is focal discontinuity in the normally hyperechoic and fibrillar architecture of the fascia, often with surrounding hypoechoic areas reflecting edema or hematoma [63] (Figure 12).

Dynamic US examination is particularly useful to assess the extent of injury, as it allows the visualization of fascial mobility and potential retraction during plantar flexion or dorsiflexion [64]. Power or color Doppler imaging may reveal increased vascularity in the surrounding tissues, indicating acute inflammation or a healing response [63,64]. Elastography can provide quantitative insights into tissue stiffness, demonstrating reduced stiffness compared to adjacent intact fascial regions [63,64].

### 3.6. Subcutaneous Tissue and Lymphatic Disorders

#### Lymphedema and Lipedema

**Figure 13:**
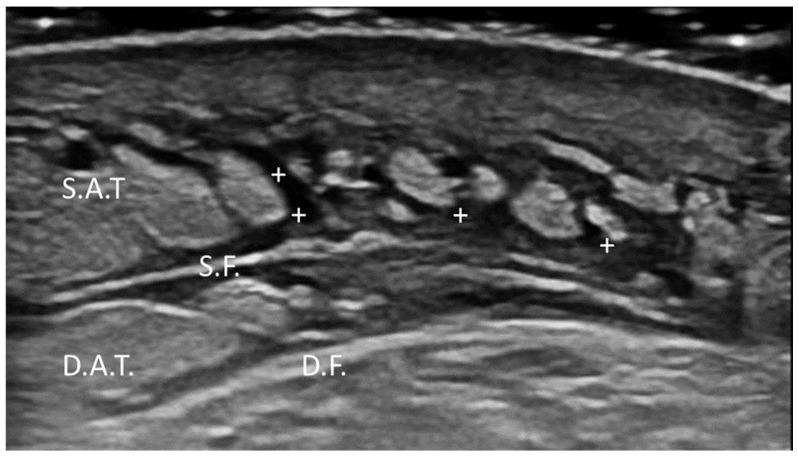
Ultrasonographic features of lymphedema in the anterior arm. US image demonstrates increased subcutaneous tissue thickness with characteristic honeycomb pattern, indicative of fluid accumulation between the retinacula cutis. +: fluid accumulation. S.F.: superficial fascia. D.F.: deep fascia. S.A.T.: superficial adipose tissue. D.A.T.: deep adipose tissue.

These are two distinct conditions frequently confused due to their overlapping clinical presentations of limb enlargement. However, these conditions differ in their etiology, tissue characteristics and response to treatment, and thus precise diagnosis is paramount. In this sense, US examination is definitely contributory [65,66,67]. Lymphedema is a chronic condition characterized by the accumulation of lymphatic fluid due to obstruction or dysfunction of the lymphatic system, eventually leading to swelling primarily in the extremities [66]. Management is based on facilitating lymphatic drainage and preventing further complications. US examination often reveals increased subcutaneous tissue thickness, with a distinctive honeycomb pattern, reflecting fluid accumulation between the retinacula cutis (Figure 13).

US examination also helps in evaluating the extension of lymphedema. Sono-palpation and dynamic examination are employed to assess compressibility, whereby lymphedematous tissue would be partially compressible, often with a slow return to its baseline shape due to high fluid content [16]. Doppler US is useful to exclude venous insufficiency in the differential diagnosis. Elastography shows increased stiffness in later stages (due to fibrosis) and less resistance to compression in acute stages.

**Figure 14:**
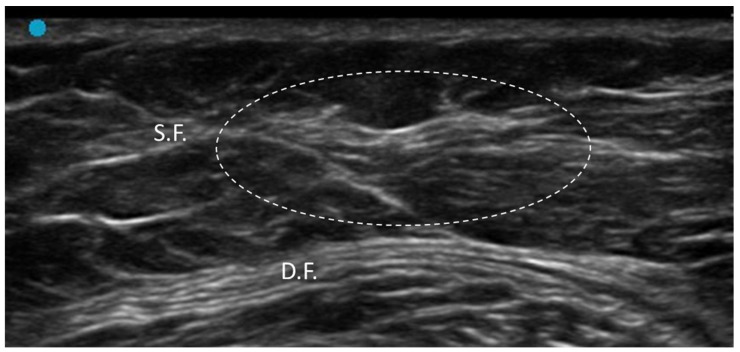
US image shows lipedema with increased subcutaneous tissue thickness and hypoechoic nodules, indicating lipomatous hypertrophy and thickened superficial fascia. S.F.: superficial fascia. D.F.: deep fascia. Dashed-line circle: thickened superficial fascia.

Lipedema, on the other hand, is a chronic adipose tissue disorder marked by abnormal fat deposition, sparing the feet and involving the thighs/buttocks. It predominantly affects women and is often triggered by hormonal changes such as puberty or pregnancy [68]. Lipedema is characterized by disproportionate accumulation of fat, leading to pain and easy bruising [68]. US examination shows increased subcutaneous fat thickness, with hypoechoic nodules representing lipomatous hypertrophy. The fasciae remain well defined, with increased thickness of the superficial fascia, superficial and deep adipose tissues [68] (Figure 14).

Dynamic US examination can show retained fat lobules and retinacula cutis, highlighting stable adipose hypertrophy [68]. Doppler US shows unremarkable findings, as there is no inflammatory hyperemia or venous insufficiency. Elastography demonstrates soft fat consistency. Sono-palpation reveals its non-pitting nature as well as noncompressible superficial/deep adipose tissue.

## 4. Discussion

The adoption of US imaging in the examination of fasciae is increasing among physicians, with its applications expanding across various medical fields, like physical and rehabilitation medicine, rheumatology, orthopedics, anesthesiology, pain medicine and sports medicine [1]. US imaging is a unique outcome measurement instrument because it is capable of identifying both structural damage and the inflammatory state [1,2,3]. Notably, the anatomy of fasciae is intricate and fundamental to understanding the biomechanics of the human body. Fasciae are dense connective tissue structures that encase and penetrate muscles, bones, nerve and blood vessels, creating a continuous, integrated network that supports and coordinates bodily functions. Superficial and deep fasciae can be distinguished both anatomically and “ultrasonographically”. The former appears as a thin, less-defined echogenic line in the subcutaneous tissue, whereas the latter is visualized as thicker, multilayered, echogenic structures with clear separation between the layers [67].

Unlike traditional imaging techniques, which often fail to correlate with the specific localization of pain, US imaging provides a multiparametric approach including dynamic assessment. It enables the visualization of fascial thickness, echogenicity, stiffness, architectural organization, deformation, shear strain and displacement, eventually providing comprehensive insights as regards the clinical scenario. This capability is invaluable in identifying fascial pathology and dysfunction that may not be apparent during physical examination [1,69,70].

In recent years, this technological advancement has coincided with a paradigm shift in the understanding of the functional role of fasciae. Increasing evidence suggests that fasciae are not merely passive structures but play an active role in proprioception, force transmission and nociceptive signaling. Therefore, pathological alterations in fasciae (such as fibrosis, increased stiffness, etc.) have been implicated in a variety of clinical conditions, ranging from chronic musculoskeletal pain to post-surgical complications. The ability of US examination to capture such fascial alterations is of paramount importance in clinical settings. Beyond qualitative observation, recent studies have supported the use of quantitative ultrasonographic parameters to objectively assess fascial morphology and biomechanics. Moreover, emerging protocols have focused on defining normative values for fascial thickness and stiffness across different anatomical regions, age groups and sexes, enhancing the clinical applicability of US fascial examination.

Another innovative application of US imaging is the assessment of fascial gliding. Normal fascial mobility ensures the harmonious movement of other anatomical structures, such the muscles. Impaired fascial gliding due to adhesions, scar tissue or inflammation can result in mechanical dysfunction and pain. High-resolution ultrasonography, especially when combined with speckle-tracking techniques or Doppler-based motion analysis, enables the in vivo measurement of fascial displacement and shear strain during dynamic movement. Recent investigations have highlighted the potential of these dynamic assessments in detecting subclinical fascial dysfunction that may not be evident during physical examination or static imaging [58,71,72].

Furthermore, the clinical impact of fascial US imaging extends to the interventional domain. US-guided procedures targeting fasciae, such as hydro-dissection, percutaneous fascial release, fascial biopsy, fascial blocks and fascial plane injections, are gaining increasing attention [73,74,75]. Beyond diagnostic and interventional applications, US imaging is proving to be an invaluable tool that is not limited to pathological conditions. Several research groups have explored its application in sport science, investigating the impact of training on fascial properties [76,77]. These studies suggested that US imaging may serve as a non-invasive tool for optimizing athletic performance and preventing injuries by monitoring fascial adaptation ton mechanical load [9,78].

Lastly, the widespread accessibility, cost-effectiveness and safety profile of US imaging make it an ideal modality for large-scale screening and longitudinal studies on fascial health. Unlike MRI or CT, ultrasonography does not involve ionizing radiation and can be repeated multiple times without risk, allowing for the dynamic assessment and monitoring of fascial properties in both clinical and research contexts.

## 5. Conclusions

In conclusion, US is a “game changer” concerning fascia examination. After initial static/dynamic B-mode scanning, Doppler imaging and elastography can also be applied for further assessment. Based on the clinical and US findings, prompt management of patients with fascia pathologies is then possible.

## Data Availability

The data presented in this study are available upon request from the corresponding author. The data are not publicly available due to privacy.
